# The Fourth War Christmas

**Published:** 1917-12-15

**Authors:** 


### THE FOURTH WAR CHRISTMAS.

### Its Celebration in some Representative Institutions.

THE celebration of Christmas 1917, surrounded as it is with difficulties caused by the general shortage in all sections of the staff, the necessity for economy in connection with food and other commodities, and the scarcity in the supply of many articles, has presented a problem to the managers of our hospitals and institutions. A few institutional authorities were exercised in their minds as to what form and extent these festivities should take this year, but generally sympathy and common sense have won through the kill-joy element. We are indebted to the authorities from institutions up and down the country for information of what is in contemplation this Christmas We hope it may prove of service as a guide to some. We are looking forward to the descriptions of the actual festivities at our hospitals this year, which should be delivered at 28 and 29 Southampton Street, Strand, London, W.C. 2, not after January 1, 1918, at the latest.

### LONDON HOSPITALS.

At King's College Hospital Christmas will be observed with great simplicity and the exercise as far as possible of strict economy. At the same time the staff have determined to make the festival as cheerful a one for the patients as their efforts can secure. The same restraint will be exhibited at Middlesex Hospital, where the festivities are to be on a very moderate scale. The children will have a Christmas Tree, and there will be a concert for the patients. The Food Controller's wishes will be carefully observed as regards diet. At University College Hospital the civil patients will be given Christmas fare, and the 250 soldiers under treatment in the hospital will have a special dinner provided by the kindness of an anonymous donor. Friends interested in the hospital have undertaken to give entertainments for all the patients, and the children are to have a Christmas Tree on Boxing Day. Despite this programme the authorities state that Christmas will be celebrated very quietly. The Seamen's Hospital Society are taking great pains to make Christmas for the seamen as nearly like the old days of the Yule Log as possible. The whole hospital will be decorated and a present provided for each patient out of a fund in charge of the matron. On Christmas Eve the choir of Greenwich Parish Church, consisting of some twenty-eight male voices, will sing carols in the corridors of the hospital, a musical treat which has always been greatly appreciated. The inner man will not be forgotten on Christmas Day, and turkeys will be included in the dinner, which is always a most cheery function at this institution. Here the sailors recall the many different parts of the globe where they have eaten previous Christmas dinners, and the customs and various viands of each. In the afternoon a special tea is provided for the patients and their friends, which is served in the wards and dining-rooms. This is to be followed by a concert given by the nursing staff, and on Boxing Day an entertainment is to be given by a well-known elocutionist. At the British Home and Hospital for Incurables, Streatham, Christmas will be celebrated in the ordinary way as far as possible.

### PROVINCIAL INSTITUTIONS.

The motto for Christmas Day at the Queen's Hospital, Birmingham, is "Make the patients happy," so far as this can be achieved with due regard to patriotic economy. The sisters are doing their best to make the wards look as cheerful as possible, and are using decorations which were saved from last year. The festivities begin at nine o'clock on Christmas morning, when the Lord Mayor and Lady Mayoress visit all the wards. Each of the male patients is afterwards allowed to smoke, cigarettes and tobacco being provided, while the women and child patients will be given chocolate where the nature of their illness permits this indulgence. Dinner is to consist of roast beef and a pudding—not a Christmas pudding, owing to the difficulty of obtaining the ingredients and their enhanced cost. In the evening an entertaiment will be given in one of the wards by the resident medical staff. The festivities are continued on New Year's Day, when a useful gift, in most cases an article of clothing costing on an average about 4s. each, is given to each patient. The cost of the presents and festivities is met by donations from many good friends of the hospital to its Christmas Fund. This year the number of patients to be provided for is increased by 132 soldiers. The nursing staff have their dinner on Christmas Day directly after the patients, and if turkeys can be bought at a reasonable price these will be included. At Addenbrooke's Hospital, Cambridge, a suitable Christmas dinner and tea will be given to the patients, but no money is to be spent on decorations or entertainments. Such of the former as have been left from past years will be utilised to give the wards a bright appearance, together with any flowers or plants sent by friends as gifts. Entertainments will be thankfully accepted as well as contributions towards providing Christmas fare for the patients on modest and inexpensive lines. The nursing staff will do their utmost to make Christmas the festival it should be, and on Christmas Eve will sing carols in procession, carrying fairy lanterns on sticks through all the darkened wards.

### ENTERTAINMENTS, BUT NO REFRESHMENTS.

At the Royal Infirmary, Liverpool, the sisters have unanimously and spontaneously foregone their Christmas dinner. The patients are to have roast beef instead of the usual turkey, and the Christmas puddings are being made according to a combination of official recipes, dates being used in place of dried fruits. At this hospital no expenses are ever incurred by the sisters in connection with decorations, the entire cost of these, and of the entertainments, being defrayed by a special fund provided by the committee, hon. staff, and other friends of the hospital. Out of this fund, too, gifts for the patients are bought; this year it is hoped the gifts, though smaller, will still be useful, whilst those for the 102 sailor and soldier patients will be the best possible. Ward decorations are to be limited to lamp-shades and a few flowers. The resident medical and nursing staffs will give concerts in each ward on Christmas Day, and during the week entertainments will be provided by friends from outside the hospital, but no refreshments will be given. At the Royal Southern Hospital, Liverpool, a special appeal has been made to the committee, medical staff, and other friends for contributions to the Christmas Festivities Fund, which has produced £100. This will enable a serviceable gift to be provided for each person in the institution, and much in the way of extra provisions for the regulation Christmas fare. Indeed, practically everything extra for Christmas is provided without recourse to the funds of the hospital. Sausages and eggs for breakfast have been promised for the staff and patients, a pair of gloves for each sister and nurse, twelve turkeys, a large quantity of best butter, and whist and billiard prizes for the military patients. The military patients are to have turkey, bread sauce, vegetables, Christmas pudding, sweets and dessert, and a pint of beer each, whilst the civilian patients will have roast beef instead of turkey, no sweets, dessert, or beer, and jellies and trifle for those who cannot eat Christmas pudding. The medical and nursing staffs have the same dinner as the military patients, but no beer. The Christmas puddings have been made with apricots, prunes, peaches, and apples in place of currants, sultanas, etc.

Christmas at the Royal Victoria Infirmary, Newcastle, will as far as possible be celebrated in the same way as hitherto. It is rather doubtful if it will be possible to provide turkey for the Christmas dinner, but this will be done if it is procurable. The Christmas puddings are being madefrom the Food Controller's recipe. The entertainments will be carried out as usual. The authorities at Norwich seem confident that the patients will have a good time this Christmas, notwithstanding necessary curtailment in the usual Christmas fare. No public appeal has been made for funds for extra comforts, but a generous friend of the hospital has undertaken to provide, as in former years, turkeys sufficient for all the patients, so that a real Christmas dinner will be possible. There will be the usual service in the chapel in the morning, a concert in the recreation hall in the afternoon, and a happy evening in the wards. On Boxing Day a theatrical performance will be given and other entertainments during the week. There are to be no general decorations, though it is expected that each ward will display some visible sign of the festive season, and that the staff will continue to do their utmost to create the Christmas atmosphere amongst the patients with the success which has hitherto attended their efforts.

